# Psychiatric presentations and admissions during the first wave of Covid-19 compared to 2019 in a psychiatric emergency department in Berlin, Germany: a retrospective chart review

**DOI:** 10.1186/s12888-023-04537-x

**Published:** 2023-01-14

**Authors:** T. Goldschmidt, Y. Kippe, A. Finck, M. Adam, H. Hamadoun, J. G. Winkler, F. Bermpohl, M. Schouler-Ocak, S. Gutwinksi

**Affiliations:** Psychiatrische Universitätsklinik der Charité im St. Hedwig Krankenhaus, Große Hamburger Str. 5-11, 10115 Berlin, Germany

**Keywords:** Psychiatry, Berlin, Covid-19, Psychiatric emergency department, Police custody, Schizophrenia

## Abstract

**Background:**

Most studies agree that the Covid-19 pandemic and the subsequent lockdown had a negative impact on mental health. On the other hand, international studies have shown that psychiatric emergency departments (pED) experienced a decrease in presentations and admissions.

**Methods:**

Retrospective chart review of all pED presentations and admissions during the first wave of Covid-19 pandemic in Germany (Covid-19 period: 3/2/20 to 05/24/20) in a psychiatric hospital in Berlin compared to 1 year earlier (pre-Covid-19 period). Descriptive statistics and logistic regression were performed.

**Results:**

We observed no statistical significant changes in overall pED presentations and overall hospital admissions during the Covid-19 period compared to the pre-Covid-19 period (813 vs. 894, − 9.1%, *p* = 0.064 and (363 vs. 437, − 16.9%, *p* = 0.080 respectively). In the subgroup analysis, less patients with depressive disorders (*p* = 0.035) and with personality disorders (*p* = 0.002) presented to the pED, a larger number of presentations with schizophrenia was observed (*p* = 0.020). In the Covid-19 period, less patients with substance use disorder and paranoid schizophrenia were admitted to the hospital via the pED than in the pre-Covid-19 period (*p* = 0.035 and *p* = 0.006, respectively). Bed capacity was reduced in the Covid-19 period by − 32.8% (*p* <  0.001).

Presentations in police custody were 13.7% (*p* = 0.029) higher during the Covid-19 compared to pre-Covid-19 period, with higher rates in female presentations (*p* = 0.008) and suicide attempts (*p* = 0.012) and less hospital admissions (*p* = 0.048). Logistic regression analyses revealed that positive predictors for pED presentation during Covid-19 period were police custody (*p* <  0.001), being redirected from another hospital (*p* <  0.001), suicide attempt (*p* = 0.038), suicidal thoughts (*p* = 0.004), presentation with paranoid schizophrenia (*p* = 0.001) and bipolar and manic disorders (*p* = 0.004), negative predictors were hospital admission (*p* <  0.001), depressive disorders (*p* = 0.021) and personality disorders (*p* <  0.001).

**Conclusions:**

A larger number of presentations in police custody during the Covid-19 period may represent untreated medical needs. This was seen predominantly in female patients, suggesting this subgroup might have suffered particularly under lockdown measures. Patients with paranoid schizophrenia were the only subgroup, which increased in absolute numbers, also suggesting a particular lockdown effect. Reduced bed capacity due to infection curbing measures is suggestive to have played an important role in augmenting the threshold for hospital admissions.

**Supplementary Information:**

The online version contains supplementary material available at 10.1186/s12888-023-04537-x.

## Introduction

Worldwide, there is a growing body of evidence suggesting that the appearance of Covid-19 and the measures to limit its propagation have had a negative impact on mental health, with as a result increased symptoms of depression and anxiety in the general population [1–3]. Certain subgroups, such as health care professionals [4, 5] or those with preexisting mental health conditions [6–8] seem to be at particular risk of adverse mental health outcomes. In psychiatric emergency departments (pED), however, attendances decreased dramatically during the first wave of Covid-19 compared to previous years, which was shown for example in a grand scale cross-sectional study in the US [9] and similar findings have been reported for Australia [10–12], France [13], Italy [14–16], New Zealand [17], Portugal [18], Spain [19, 20], Switzerland [21], Turkey [22], the UK [23] and the US [24]. Decreases range from 4% [25] to 56% [14]. Regarding Germany there are three studies on pED presentations so far. One study from Mannheim focused on the first 7 weeks of the first wave of Covid-19, stating a relation between reduced general mobility and the decrease of pED presentations with affective disorders [26]. A rather small study from Munich with 57 psychiatric presentations in an 8 weeks observation period did not find a change in pED presentations during the first Covid-19 wave [27]. A study from Hannover showed a 21.4% decrease during the first Covid-19 wave compared to 2019 [28]. On the first glance this worldwide decrease in pED attendances during the first wave of Covid-19 compared to previous years does not quite seem to fit the above-mentioned negative impact on mental health. It was hypothesized that several factors may have caused this decrease such as a moral conscience to not overload emergency facilities [18] leading to a higher threshold of symptom severity until help is requested [23]. The fear to get infected at a pED has been suggested as the most important factor [23, 29, 30].

While the number of pED presentations decreased, an increase of severity has been observed [14, 19, 21] during the first wave of Covid-19 in comparison to previous years: Montalbani et al. calculated a severity score based on psychopathological status of patients indicating an increase in severity [14]. Other authors report a change in diagnostic groups towards more severe mental illnesses [19] and an increase in suicidal tendencies and referrals by ambulance [21]. There are studies, which do not report an increase of more severe cases during Covid-19 pandemic: Dragovic et al. found in a large sample of *N* = 7140 pED presentations in Australia a lower number of suicidal behavior during the first wave of Covid-19 [11]. Furthermore, most studies show a general decrease in hospital admissions [31, 32], ranging from − 12% [21, 33] to − 48% [18] – only one study found an increase in admissions by 8% [11].

In summary, there are heterogenous findings until date regarding pED presentations and admissions during the first wave of Covid-19. These may be due to differences in Covid-19 infection rates between countries and the subsequent governmental response which differ between countries [34]. The extent of these responses with varying restrictions to daily life may be linked to the degree of the negative impact to mental health [35]. Differences in reported severity, however, can also be appreciated between studies in the same country as shown in Italy [14, 15, 31]: the group of Montalbani et al. reports an increase in suicidality [14], whereas the group of Beghi et al. see a decrease during the first wave of Covid-19 [15]. This stresses the impact of local sociodemographic differences and potentially of the local perception of danger [36].

Due to the federal structure of German policymaking, various lockdown measures were put into effect at different times. The following dates are valid for Berlin: from March 12th 2020 on, schools were closed down; universities were recommended to implement digital education formats [37]. In a next step, cultural and sport facilities such as theatres, cinemas, discotheques, bars, swimming pools, fitness studios etc. as well as all non-vital businesses had to shut down. Furthermore, religious gatherings in churches, synagogues and mosques were prohibited [38]. The third round of lockdown restrictions were put into place March 22nd 2020: from that date on, a minimum distance of 1.5 m to persons outside the own household in public places had to be held. It was furthermore prohibited to meet more than one person outside the own household in public as well as in private (contact bans). At this point, also gastronomy and other services had to close except for take-away food [39]. These restrictions led to reduced mobility in the general population and has been correlated to a reduction in psychiatric emergency presentations during the first weeks of the Covid-19 pandemic in Mannheim, Germany [26]. It seems plausible that this was also the case in Berlin. The psychiatric healthcare system was also affected by attempts to reduce infections: in our clinic, there was a significant reduction in bed capacity (Table 1), in the outpatient sector, telemedical consulting methods were implemented. In Germany, the perception of patients having an ensured medical access was lower during the first lockdown (86.8%) compared to later lockdowns (94.1% during second lockdown) in an online survey in the general population [40], no data is available on psychiatric patients alone. In psychiatric hospitals, strict restrictions for visiting patients were implemented and many outpatient and day-clinical treatment offers were shut down. This resulted in a decreased availability of treatment options for many psychiatric patients [41].

The first measures to be lifted were those regarding schools, as of the 4th of May 2020 elementary schools and graduate classes were reopened [42]. Shortly after, the contact bans were loosened to a degree that members of two households were allowed to meet in public. Also from this point on, a hot spot policy was put into place, which allowed for local lockdown measures in places of high incidences [43].

## Methods

### Study design

We conducted a retrospective chart review of clinical documentation records of all presentations at the psychiatric department at St. Hedwig Hospital (SHK) in Berlin - a part of the Charité University Psychiatry department - during the first wave of the Covid-19 pandemic in Germany. The study was approved by the local ethics committee (Ethikkommission der Charité – Universitätsmedizin Berlin; number of approval: EA 110/20).

This study focuses on a 12-week period from 03-02-2020 to 05-24-2020 and the same period 1 year earlier, examining presentations and admissions in a psychiatric emergency department (pED) in Berlin, Germany. Parameters of interest were the rates of pED presentations and admissions, age, gender, homelessness, Covid-19 high-risk group, the mode of attendance (in police custody, redirected from other hospital, self-referring, with family/friends, with legal guardian/social worker, ambulance/paramedics), clinical characteristics (diagnostic groups, suicidal tendencies, aggressive behavior), the subgroup coming in police custody and the subsequent fate of the patient (discharge, admission, voluntary, involuntary).

**Fig. 1:**
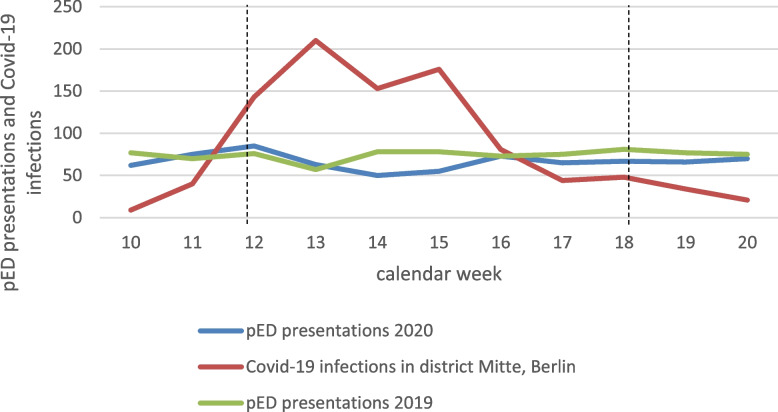
pED presentations and Covid-19 infections in district Mitte, Berlin. Displays weekly pED presentations in the Covid-19 period (2020, blue line) and pre-Covid-19 period (2019, green line) and weekly Covid-19 infection numbers in the pED’s catchment area in Berlin (red line) in the calendar weeks 10–20. No difference in pED presentations in the Covid-19 period compared to the pre-Covid-19 period was seen. Weeks 9 and 21 are not displayed, as the observation periods did not include complete weeks in both years. The two vertical dotted lines indicate the beginning of lockdown measures in Germany (left line; 03/22/2020) and first opening steps (right line, 05/04/2020) respectively. Data on Covid-19 infections from Robert-Koch-Institut: https://survstat.rki.de/Content/Query/Create.aspx; Abbreviations used: *pED, *psychiatric emergency department

The Covid-19 period covers the entire first wave of Covid-19 in Berlin: the beginning of the observation period (03-02-2020) is marked by the date of the first publicly known Covid-19 case in Berlin [44]; it ends with the date of the curb reaching the bottom number of newly registered cases of Covid-19 infections in Berlin (05-24-2020), marking the end of the first wave [45] (cf. Fig. 1). The comparison period (“pre-Covid-19 period”) was defined as the same period 1 year earlier, i.e. the 2nd of March to 24th of May 2019.

The psychiatric department of SHK consists of an emergency department and 7 psychiatric care units for inpatient treatment (bed capacity: 149). The psychiatric hospital is responsible for residents (and homeless people) of the districts Moabit, Tiergarten and Wedding in Berlin, which make up a catchment area of approximately 327.000 people. However, a large amount of people attending the emergency department live outside the catchment area (cf. Table 1). The SHK psychiatric department is obliged to provide inpatient treatment for patients (residents and homeless) living within the above-mentioned districts and having an indication for inpatient treatment, whereas patients who live in other districts of Berlin are usually redirected to the psychiatric department of their district when inpatient treatment is needed.

In the Covid-19 period one of the seven wards was turned into a Covid-19 ward, isolating psychiatric patients with confirmed or suspected Covid-19 infection. In addition, when possible, on the other wards newly admitted patients were no longer admitted in shared but only in single rooms and were transferred in shared rooms at the earliest after a negative PCR result (normally 2 days after admission). These interventions and guidelines reduced the admission capacity. We possess of monthly data on bed utilization statistics for 2018, 2019 and 2020. In the months March to May in 2018 and 2019, on a monthly scale, at least 96% of available beds were used for admissions (data not shown). We assume, from clinical experience, that in 2020 most available beds were used for admission with as sole exception the Covid-19 ward (16% of all available beds in non-Covid period). Thus, we consider the bed utilization statistics as a good approximation of total bed capacity for both the pre-Covid-19 and the Covid-19 period.

Cases were excluded if they concerned scheduled admissions to psychiatric wards or admissions to a day therapy unit. The latter were shut down during the beginning of the pandemic. Additional exclusion criteria were: patients who left without being seen by a psychiatrist or when no psychiatric F-diagnosis according to the International Statistical Classification of Diseases and Related Health Problems, 10th revision (ICD-10) was documented.

We assessed different attendance modes to the pED (Table 1) as this may give information about acuity. We defined a group of patients having increased risk for a severe form of Covid-19 (Covid-19 high-risk group) according to the then known risk factors (such as obesity, age, cardiovascular diseases, lung diseases and severe other medical conditions including cancer [46]), as this may have an influence on anxiety and perceived stress.

Considering high frequent attenders and the possible bias this group would impose on our results, we merged inpatient stays, if they were separated by less than 3 days. If cases were separated by 4–7 days, they were only merged if discharge was because of somatic complications or against documented advice of medical staff. An overview of excluded cases can be found in supplementary material S1.

For analysis of our data, we grouped cases into 7 different diagnostic categories: Organic mental disorders (OMD), substance use disorders (SUD), schizophrenia and psychotic disorders (SPD), bipolar and manic disorders (BMD), depressive disorders (DD), neurotic-, somatoform-, and stress-related disorders (NSD) and personality disorders (PD). OMD consists of ICD-10 diagnoses F00-F09, SUD of ICD-10 diagnoses F10-F19 except for nicotine related disorders (F17) and substance related psychotic disorders (F1x.5 and F1x.7). The latter are next to ICD-10 diagnoses F20-F29 part of the SPD group. BMD is composed of ICD-10 diagnoses F30-F31, DD of F32-F33. NSD is a group made up of all F40-F48 diagnoses, PD contains diagnoses F60-F62. We considered the principal diagnosis and all secondary diagnoses for the classification since the principal diagnosis was not always clearly distinguishable. This allows for the possibility of one case being in multiple diagnostic categories.

Additionally, we defined certain diagnoses of special interest derived from clinical experience, to investigate their role in diagnostic categories. These are: paranoid schizophrenia (F20.0) in context of SPD, severe forms of depression (F32.2, F32.3, F33.2, F33.3) in context of DD.

### Statistical analysis

Quantitative variables were tested for normal distribution utilising the Kolmogorov-Smirnov-Test and via graphic examination of the Q-Q-Plot. Since all quantitative variables were not normally distributed, only medians are reported; for description of qualitative variables, absolute numbers and percentages are presented. Comparisons of medians between groups were performed using the Mann-Whitney-U-Test, comparisons of percentages between groups were performed using the Chi^2^ test unless otherwise stated. For statistical tests where expected case numbers were lower than 5, the Fisher-exact-test was used. The *p*-value for statistical significance was set to *p* <  0,05.

We fitted a hierarchical 2-step logistic regression model with main and interaction effects to estimate effect sizes of a range of regressors derived from literature on the subject. As outcome variable, we chose “Covid-19 period” vs “pre-Covid-19 period”. All statistical analyses were performed using the SPSS statistical package, version 27.0, IBM Corporation (2020), (RRID:SCR_002865).

## Results

Our data consists of a total of *N* = 2314 psychiatric emergency department (pED) and admission files which were documented during the two observation periods. After applying our exclusion criteria, of 1707 cases, 813 cases remained for the Covid-19 period and 894 cases for the pre-Covid-19 period (Table 1, Fig. 1), indicating a decline in total pED attendances by − 9.1% when comparing the Covid-19 to the pre-Covid-19 period. The mean rate of weekly pED presentations was − 9.1% lower in the Covid-19 period compared to the pre-Covid-19 period, though not statistically significant (67.75 vs. 74.50, *p* = 0.064, Table 1). Between the Covid-19 and the pre-Covid-19 period no statistically significant differences were found regarding age and gender (Table 1). No differences were found regarding presentation rate of homeless (Table 1). Zero cases of admissions have been tested positive for Covid-19 during the first wave within the 5 days from admission (internal data, not shown). pED presentations who were eventually discharged, were not tested on a regular basis, no positive cases were detected.

**Table 1:** Clinical and demographic characteristics of pED presentations

	***2019 pre-Covid-19 period***	***2020 1st wave of Covid-19***	***difference between abs. numbers***	*p-value*
	cases (% of all cases in time period))	cases (% of all cases in time period))	%	
**N total number of presentations**	894	813	−9.1	
**Mean of weekly pED presentations (SD)**	74.50 (6.16)	67.75 (10.14)	−9.1	*p* = 0.064
**Median age**	39 years	39.5 years	+ 1.3	*p* = 0.077
**Female gender**	383 (42.8)	311 (38.3)	−18.8	*p* = 0.055
**Homeless**	86 (9.6)	91 (11.2)	+ 5.8	*p* = 0.287
**Covid-19 high-risk group**	231 (25.8)	224 (27.6)	−3.0	*p* = 0.424
Provenance (a)				***p*** **= 0.017**
**Berlin, within SHK catchment area**	509 (56.9)	517 (63.9)	+ 1.6	
**Berlin, outside SHK catchment area**	320 (35.8)	235 (29.0)	−26.6	
**Not Berlin, but in Germany**	56 (6.3)	52 (6.4)	−7.1	
**Residency outside Germany**	9 (1.0)	5 (0.6)	−44.4	
**No information available**	0 (0.0)	4 (0.5)	–	
Mode of attendance				***p*** **= 0.001**
**In police custody**	146 (16.3)	166 (20.4)	+ 13.7	
**Redirected from other hospital**	85 (9.5)	113 (13.9)	+ 32.9	
**Self-referring**	307 (34.3)	258 (31.7)	−16.0	
**With family/friends**	135 (15.1)	83 (10.2)	−38.5	
**With legal guardian/social worker**	27 (3.0)	24 (3.0)	−11.1	
**Ambulance/paramedics**	194 (21.7)	168 (20.8)	−13.4	
Diagnostic categories				
***Organic mental disorders***	48 (5.4)	40 (4.9)	−16.7	*p* = 0.675
***Substance use disorders***	416 (46.5)	405 (49.8)	−2.6	*p* = 0.175
***Schizophrenia and psychotic disorders (SPD)***	288 (32.2)	282 (34.7)	−2.1	*p* = 0.280
*Paranoid schizophrenia (F20.0)*	115 (12.9)	137 (16.9)	+ 19.1	***p*** **= 0.020**
*Other SPD*	173 (19.4)	145 (17.8)	−16.2	*p* = 0.422
***Bipolar and manic disorders***	48 (5.4)	58 (7.1)	+ 20.8	*p* = 0.131
***Depressive disorders (DD)***	143 (16.0)	101 (12.4)	−29.4	***p*** **= 0.035**
*Severe depression (F32.2, F32.3, F33.2, F33.3)*	59 (6.6)	38 (4.7)	−35.6	*p* = 0.086
*Other DD*	84 (9.4)	63 (7.7)	−25.0	*p* = 0.226
***Neurotic-, somatoform- and stress-related disorders (NSSD)***	156 (17.4)	143 (17.6)	−8.3	*p* = 0.940
***Personality disorders (PD)***	132 (14.8)	79 (9.7)	−40.2	***p*** **= 0.002**
Discharge/admission				p = 0.080
**Discharge**	457 (51.1)	450 (55.4)	−1.5	
**Hospital admission (admission rate)**	437 (48.9)	363 (44.6)	−16.9	
**Bed capacity (%)**	97.19	65.27	−32.8	***p*** **< 0.001**
**Admissions, rate (%) - OMD**	34 (70.8)	25 (62.5)	−26.5	*p* = 0.408
**Admissions, rate (%) - SUD**	238 (57.2)	202 (49.9)	−15.1	***p*** **= 0.035**
**Admissions, rate (%) - SPD**	180 (62.5)	151 (53.5)	−16.1	***p*** **= 0.030**
**Admissions, rate (%) - F20.0**	77 (67.0)	68 (49.6)	−11.7	***p*** **= 0.006**
**Admissions, rate (%) - BMD**	32 (66.7)	39 (67.2)	+ 21.9	*p* = 0.950
**Admissions, rate (%) - DD**	61 (42.7)	42 (41.6)	−31.1	*p* = 0.867
**Admissions, rate (%) - F32.2, F32.3, F33.2, F33.3**	31 (52.5)	20 (52.6)	−35.5	*p* = 0.993
**Admissions, rate (%) - NSSD**	47 (30.1)	37 (25.9)	−21.3	*p* = 0.414
**Admissions, rate (%) -PD**	65 (49.2)	38 (48.1)	−41.5	*p* = 0.872
**Median length of admission - all cases**	10 days	8 days	−20.0	***p*** **= 0.007**
Legal status of admission				*p* = 0.700
**Voluntary**	815 (91.2)	739 (91.7)	−9.3	
**Involuntary**	79 (8.8)	67 (8.3)	−15.2	

“Difference” is the relative change of absolute numbers in 2020 compared to 2019 in percentage values. *P*-values are resulting from chi^2^-tests, exept for “median age” and “median length of admission”, which were tested using the Mann-Whitney-U-test. Abbreviations used: N, n = case numbers; SHK = St. Hedwig hospital; F-codes refer to the International Classification of Diseases, 10th revision; (a): “no information available” was not included in statistical testing, as this would not allow for the Chi^2^-test due to low case numbers

### Covid-19 high-risk group

We ex post classified 224 (27.6%) cases of having increased risk for a severe form of Covid-19 pneumonia during the Covid-19 period, not significantly differing from the pre-Covid-19 period.

### Provenance

The provenance of presentations changed significantly between the Covid-19 and the pre-Covid-19 period (overall *p* = 0.017, Table 1). In proportion there was an increase of presentations from the districts in Berlin that our pED is responsible for (catchment area) in the Covid-19 period (63.9% vs. 56.9%; Table 1); presentations from Berlin, but outside the responsible districts were less in the Covid-19 compared to the pre-Covid-19 period (235 vs. 320, Table 1).

### Mode of attendance

Significant changes were found in mode of attendance (overall *p* < = 0.001, Table 1), characterized by a higher number of presentations in police custody in the Covid-19 period compared to the pre-Covid-19 period (166 vs. 146, *p* = 0.029, Table 2) and a higher number of patients being redirected from other hospitals (113 vs. 85). These both were also the two strongest predictors of Covid-19 period in our logistic regression model (OR: 2.440; 95% CI: 1.584–3.761; *p* <  0.001 and OR: 2.480; 95% CI: 1.647–3.736; *p* <  0.001 respectively, Table 4).

**Table 2:** Characterization of attendances in police custody

	***2019 pre-Covid-19 period***	***2020 1st wave of Covid***	***difference between abs. numbers***	*p-value*
	cases (% of all cases in police custody))	cases (% of all cases in police custody))	%	
Attendances in police custody				
**N (% of total cases)**	146 (16.3)	166 (20.4)	+ 13.7	***p*** **= 0.029**
**Median age**	35 years	37 years	+ 5.7	*p* = 0.629
**Female gender**	38 (26.0)	67 (40.4)	+ 76.3	***p*** **= 0.008**
**Homeless**	23 (15.8)	23 (13.9)	±0.0	*p* = 0.637
**Covid-19 high-risk group** (a)	32 (21.8)	39 (23.5)	+ 21.9	*p* = 0.740
Psychopathology of cases in police custody				
**Signs of delusion**	70 (52.2)	68 (48.2)	−2.9	*p* = 0.506
**Aggressive behaviour towards others**	72 (49.3)	60 (36.1)	−16.7	***p*** **= 0.019**
**Suicidal thoughts**	24 (19.4)	34 (23.4)	+ 41.7	*p* = 0.416
**Suicidal plans**	8 (6.4)	18 (12.6)	+ 125.0	*p* = 0.088
**Suicide attempts**	2 (1.4)	12 (7.5)	+ 500.0	***p*** **= 0.012**
Hospital admission after police custody				**p = 0.048**
**yes**	106 (72.6)	103 (62.0)	−2.8	
**no**	40 (27.4)	63 (38.0)	+ 57.5	
Legal status of admissions after police custody				*p* = 0.103
**Voluntary**	54 (50.9)	64 (62.1)	+ 18.5	
**Involuntary**	52 (49.1)	39 (37.9)	−25.0	

**“**Difference” is the relative change of absoulte numbers in 2020 compared to 2019 in percentage values. *P*-values are resulting from chi^2^-tests, exept for “suicide attempts”, which were tested using the Fisher-exact-test and “median age” and “median length of admission”, which were tested using the Mann-Whitney-U-test. Abbreviations used: *N* = case numbers; *Covid-19 * Coronavirus disease 2019

In the Covid-19 period we observed less self-referring (258 vs. 307, − 16.0%) and less attendances accompanied by family or friends (83 vs. 135, − 38.5%) compared to the pre-Covid-19 period, cf. Table 1. The presentations with ambulance and accompanied by guardian or social worker did not change in terms of rates to all attendances in the two observed time (20.8% vs. 21.7 and 3.0% vs 3.0%, Table 1).

### Differences in diagnostic categories

The median number of diagnostic categories per attendance was 1, no differences were found between the Covid-19 and the pre-Covid-19 period (*p* = 0.727; Z = − 0.350). We found different effects regarding changes in pED attendance numbers across different diagnostic categories (Table 1): the most prominent difference in numbers was observed in the group of personality disorders (PD), with 40.2% (*p* = 0.002) less presentations when comparing the Covid-19 with the pre-Covid-19 period. In the group of depressive disorders (DD), 29.4% (*p* = 0.035) less presentations were observed in the Covid-19 period. This was seen in both, severe forms of depression and more mild forms (Table 1). In the logistic regression model, both PD and DD were negatively related to Covid-19 period (OR: 0.539; 95% CI: 0.383–0.758; *p* < 0.001 and OR: 0.694; 95% CI: 0.509–0.947; *p* = 0.021, respectively, Table 4). Presentations with schizophrenia and other psychotic disorders (SPD) had 3.0% did not change between the two time periods (Table 1). When focusing on paranoid schizophrenia alone, which was the most often diagnosed psychotic disorder, we even saw 19.1% higher absolute numbers (*p* = 0.020, Table 1). In the logistic regression model, paranoid schizophrenia was a predictor for pED attendance in the Covid-19 period (OR: 1.712; 95% CI: 1.239–2.367; *p* = 0.001, Table 4). The group of bipolar and manic disorders (BMD) with non-significantly higher absolute case numbers of 20.8% was also a predictor for pED attendance in the Covid-19 period (OR: 1.946; 95% CI: 1.240–3.054; *p* = 0.004, Table 4). The groups of organic mental disorders (OMD), substance use disorders (SUD) and neurotic, somatoform, and stress-related disorders (NSD) were not differing significantly.

### Changes in police custody

**Fig. 2:**
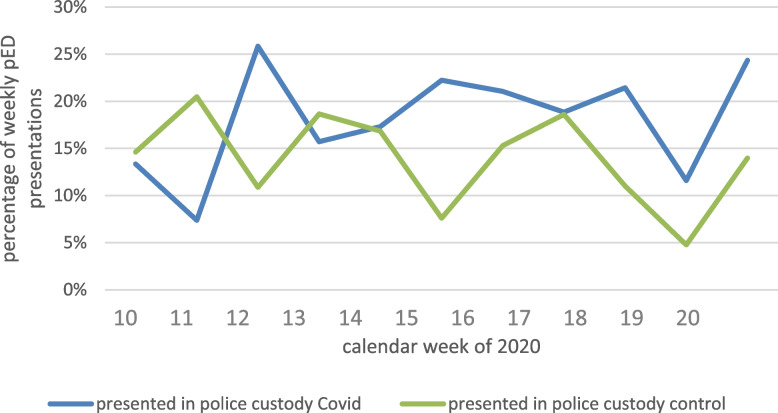
pED presentations in police custody. Displays the percentage of pED presentations in police custody per week per observed time period: the blue line (Covid-19 period, 2020) is mostly above the green line (pre-Covid-19 period, 2019), in particular from week 15 on (beginning of April). Abbreviations used: *pED,* psychiatric emergency department

When focusing on the subgroup of cases attending the pED in police custody, we found more female cases being presented in police custody in the Covid-19 period compared to the pre-Covid-19 period (67 vs. 38, *p* = 0.008, Table 2). Fig. 2 shows the rate of presentations in police custody per year.

In the subgroup of cases brought by police, the number of reported signs of delusion, suicidal thoughts and suicidal plans did not differ between the Covid-19 and the pre-Covid-19 period (cf Table 2). The number of suicidal attempts prior to the pED attendance was significantly higher in the Covid-19 period than in the pre-Covid-19 period (12 vs. 2, *p* = 0.012, Table 2). Within the subgroup of presentations in police custody, reported aggressive behaviour towards others was significantly less often in the Covid-19 period (60 vs. 72, *p* = 0.019, Table 2). Rates of hospital admissions after presentation in police custody were significantly lower in the Covid-19 period than in the pre-Covid-19 period (62.0% vs. 72.6%, *p* = 0.048, Table 2). No difference was seen in legal status of admissions after presentation in police custody (*p* = 0.103, Table 2).

We saw significantly more attendances in police custody to the pED during the Covid-19 period with a diagnosis of personality disorder when compared with the pre-Covid-19 period (23 vs. 22, *p* = 0.033, Table 3). There were no differences in attendances in police custody between both periods concerning other diagnostic groups (Table 3). A trend to more attendances in police custody to the pED during the Covid-19 period was observed in patients with paranoid schizophrenia (40 vs. 22, *p* = 0.065). There were no differences between both periods in involuntary admissions subsequently to attendances in police custody across all assessed diagnostic groups (Table 3).

**Table 3:** Attendances in police custody and subsequent involuntary admissions across diagnostic categories

	***2019 pre-Covid-19 period***	***2020 1st wave of Covid***	***difference between abs. Numbers***	*p-value*
	cases (% of cases with respective feature)	cases (% of cases with respective feature))	%	
**Organic mental disorders**				
Attendance in police custody	11 (22.9)	5 (12.5)	−54.5	*p* = 0.207
involuntary admission	9 (18.8)	4 (10.0)	−55.6	*p* = 0.249
**Substance use disorders**				
Attendance in police custody	95 (22.8)	95 (23.5)	±0.0	*p* = 0.833
involuntary admission	44 (10.6)	40 (9.9)	−9.1	*p* = 0.741
**Schizophrenia and psychotic disorders**				
Attendance in police custody	64 (22.2)	80 (28.4)	+ 25.0	*p* = 0.091
involuntary admission	46 (16.0)	32 (11.3)	−30.4	*p* = 0.108
**Paranoid schizophrenia**				
Attendance in police custody	22 (19.1)	40 (29.2)	+ 81.8	*p* = 0.065
involuntary admission	19 (16.5)	19 (13.9)	±0.0	*p* = 0.558
**Bipolar and manic disorders**				
Attendance in police custody	10 (20.8)	16 (27.6)	+ 60.0	*p* = 0.421
involuntary admission	7 (14.6)	12 (20.7)	+ 71.4	*p* = 0.415
**Depressive disorders**				
Attendance in police custody	5 (3.5)	5 (5.0)	±0.0	*p* = 0.573
involuntary admission	2 (1.4)	1 (1.0)	−50.0	*p* = 1.000
**Neurotic-, somatoform- and stress-related disorders**				
Attendance in police custody	11 (7.1)	18 (12.6)	+ 63.6	*p* = 0.106
involuntary admission	3 (1.9)	0 (0.0)	–	*p* = 0.249
***Personality disorders***				
Attendance in police custody	22 (16.7)	23 (29.1)	+ 4.5	***p*** **= 0.033**
involuntary admission	10 (7.6)	7 (8.9)	−30.0	*p* = 0.740

“Difference” is the relative change of absolute number in 2020 compared to 2019 in percentage values. *P*-values are resulting from chi^2^-tests, exept for “involuntary admission” of cases with depressive disorders and cases with neurotic-, somatoform- and stress-related disorders, which were tested using the Fisher-exact-test

### Suicidality

Logistic regression revealed suicidal thought and suicide attempt prior to pED presentation to be a predictor of Covid-19 period (OR: 1.517; 95% CI: 1.141–2.017; *p* = 0.004 and OR: 1.961; 95% CI: 1.037–3.708; *p* = 0.038, Table 4).

**Table 4:** Results from the hierarchical logistic regression model estimating predictors for “Covid-19 period” vs. “pre-Covid-19 period”

	Exp (B)	95% CI lower	95% CI upper	*p*-value
**Age**	1.004	0.997	1.011	0.307
**Gender**	0.949	0.764	1.181	0.641
**SHK catchment area**	1.002	0.991	1.012	0.763
Mode of attendance				
**Self-referred**	0.948	0.697	1.288	0.732
**Redirected**	2.480	1.647	3.736	**< 0.001**
**Ambulance**	1.267	0.896	1.792	0.181
**In police custody**	2.440	1.584	3.761	**< 0.001**
Psychopathology				
**Aggressive behaviour**	1.416	0.778	2.577	0.255
Aggressive behaviour*police	0.465	0.212	1.022	0.057
Suicidality				
**Suicidal thoughts**	1.517	1.141	2.017	**0.004**
**Suicidal plans**	1.515	0.968	2.370	0.069
**Suicide attempt**	1.961	1.037	3.708	**0.038**
Diagnostic categories				
**Organic mental disorders**	1.034	0.605	1.766	0.903
**Substance use disorders**	1.033	0.816	1.308	0.788
**Paranoid schizophrenia**	1.712	1.239	2.367	**0.001**
**Bipolar and manic disorders**	1.946	1.240	3.054	**0.004**
**Depressive disorders**	0.694	0.509	0.947	**0.021**
**Neurotic, somatoform and stress-related disorders**	0.990	0.741	1.322	0.946
**Personality disorders**	0.539	0.383	0.758	**< 0.001**
Admission				
**Hospital admission**	0.458	0.366	0.547	**< 0.001**
**Involuntary admission**	0.762	0.490	1.186	0.229

Odds ratios (= Exp (B)) greater than 1 indicate, that a factor is predicting a case to be within the “Covid-19 period”; odds ratios below 1 indicate, that a factor is predicting a case to be within the “control period”. Abbreviations used: *95% CI* 95% confidence interval, *SHK* St. Hedwig hospital

### Discharge and hospital admission

No significant difference between the Covid-19 and the pre-Covid-19 period regarding admission vs. discharge was seen (363 vs. 437--16.9%, Table 1). tThe number of discharged cases remained unchanged (452 vs. 457, Table 1). In the logistic regression model, hospital admission was the strongest negative predictor of Covid-19 period (OR: 0.458; 95% CI: 0.366–0.547; *p* < 0.001, Table 4). The duration of hospital treatment was significantly shorter in the Covid-19 period (8 days vs. 10 days in 2019; *p* = 0.007, Table 1).

## Discussion

To our knowledge, this is the first study examining psychiatric emergency department (pED) visits and admissions in Berlin during the first wave of the Covid-19 pandemic.

We found a no significant change in pED presentations in absolute numbers (− 9.1%, Table 1) and in weekly rates (− 9.1%, *p* = 0.064, Table 1 and Fig. 1) during the Covid-19 period compared to the pre-Covid-19 period. Most studies on pED attendances during the first wave of Covid-19 report decreases in pED presentations ranging from 15 to 40% [9, 10, 15, 17, 19, 23, 24, 26, 28, 47], mostly from Europe, America, and the Pacific region. However, some studies report even higher decreases [11, 14, 18], others as Simpson et al. report a range of 4 to 9% decreases in pED attendances in different regions of the US, partly not reaching statistical significance [25]. This underlines the impact of regional factors on pED presentations. Potential reasons for a decline in presentations will be discussed in the following paragraphs for each statistically significant difference that we found in the respective subgroups.

We saw no significant differences in age, gender and homeless status between the Covid-19 and the pre-Covid-19 period (Table 1), which is in line with most studies [21, 24, 28, 48].

### Covid-19 high-risk group

When comparing the two time periods regarding to risk factors for a severe Covid-19 infection that were known in the beginning of the pandemic, no difference in the number of pED attendances was found. One could have hypothesized that there were less self-referred presentations in the Covid-19 high-risk group during the first wave of Covid-19 for reasons of self-protection, which was not observed in our sample.

### Provenance

The rate of presentations from our catchment area increased during the Covid-19 period compared to the pre-Covid-19 period (Table 1). This, in combination with an important decrease of presentations from Berlin outside our catchment area (63.9 vs. 56.9%, Table 1), suggests a concentration to more local help-seeking behaviour and has not been shown yet in other studies focusing on pEDs. It may be due to stay-at-home advice of the government and a reduced mobility of patients [26, 49] (cf. last paragraph of Introduction). This view is supported by the observed decrease in presentations from other parts in Germany and outside Germany (Table 1). However, we also found that more cases were redirected from other hospitals in the Covid-19 period vs. the pre-Covid-19 period (Table 1), redirection being a strong predictor of Covid-19 period in the logistic regression model (Table 4). Redirection means that a patient requiring hospital admission attends a pED outside his responsible district and is subsequently transferred to the pED of his responsible clinic. Supposedly, the concentration on more local psychiatric help is also due to this mechanism. From clinical experience, we know that redirection takes place more often when admission capacities are scarce (cf paragraph on “Discharge/hospital admissions” in the Discussion).

### Mode of attendance

The decrease in self-referring attendances in the Covid-19 period in our sample is in line with other studies, which discuss that fear of infection with Covid-19 limits voluntary pED attendances [11, 21]. Attendances accompanied by family/friends decreased in our sample in the Covid-19 period compared to the pre-Covid-19 period, too. It could be discussed that this is due to social distancing measures and the contact ban during the observed Covid-19 period. A main finding of this study is that the total number and proportion of patients brought in police custody increased during the observed Covid-19 period when compared to the same period in 2019 (Table 1). This is backed by the finding that police custody was a strong predictor for Covid-19 period in the logistic regression analysis (Table 4). This finding is even more remarkable as overall case numbers decreased during the Covid-19 period. Our findings differ with previous research concerning mode of attendance, because others do not report [13, 18, 19, 49] or show no significant differences in presentations with police custody between the Covid-19 period and the pre-Covid-19 period [11, 21]. In our sample, presentations in police custody are overall more frequent compared to these studies (20.4% in the Covid-19 period, 16.3% in the pre-Covid-19 period in our study compared to 7 or 10% of presentations in police custody in other studies [11, 21]). Another difference to the studies from Switzerland and Australia is that in their samples pED presentations with ambulance did increase during the Covid-19 pandemic (from 45.4% vs. 23.3% in Switzerland [21] and 46.0% vs. 43.6% in Australia [11]). In our sample, however, there were no differences in presentations by ambulance between the pre-Covid-19 and the Covid-19 period (percentages around 20%, Table 1). The subgroup of cases in police custody in the Covid-19 period in our sample did not show more aggressive behaviour towards others but was more likely to be suicidal (Table 2). This in mind, it could be discussed that part of the increase in police custody presentations in our sample would be in other countries more likely to be seen in an increase in ambulance presentations. Further studies are needed to investigate if there are Covid-independent differences in mode of attendance of suicidal patients between countries. Also, it may be possible that other local-bound factors such as distinctions in psychiatric care systems and/or diagnostic categories of the sample determine the differences between studies.

### Diagnostic categories

**Fig. 3:**
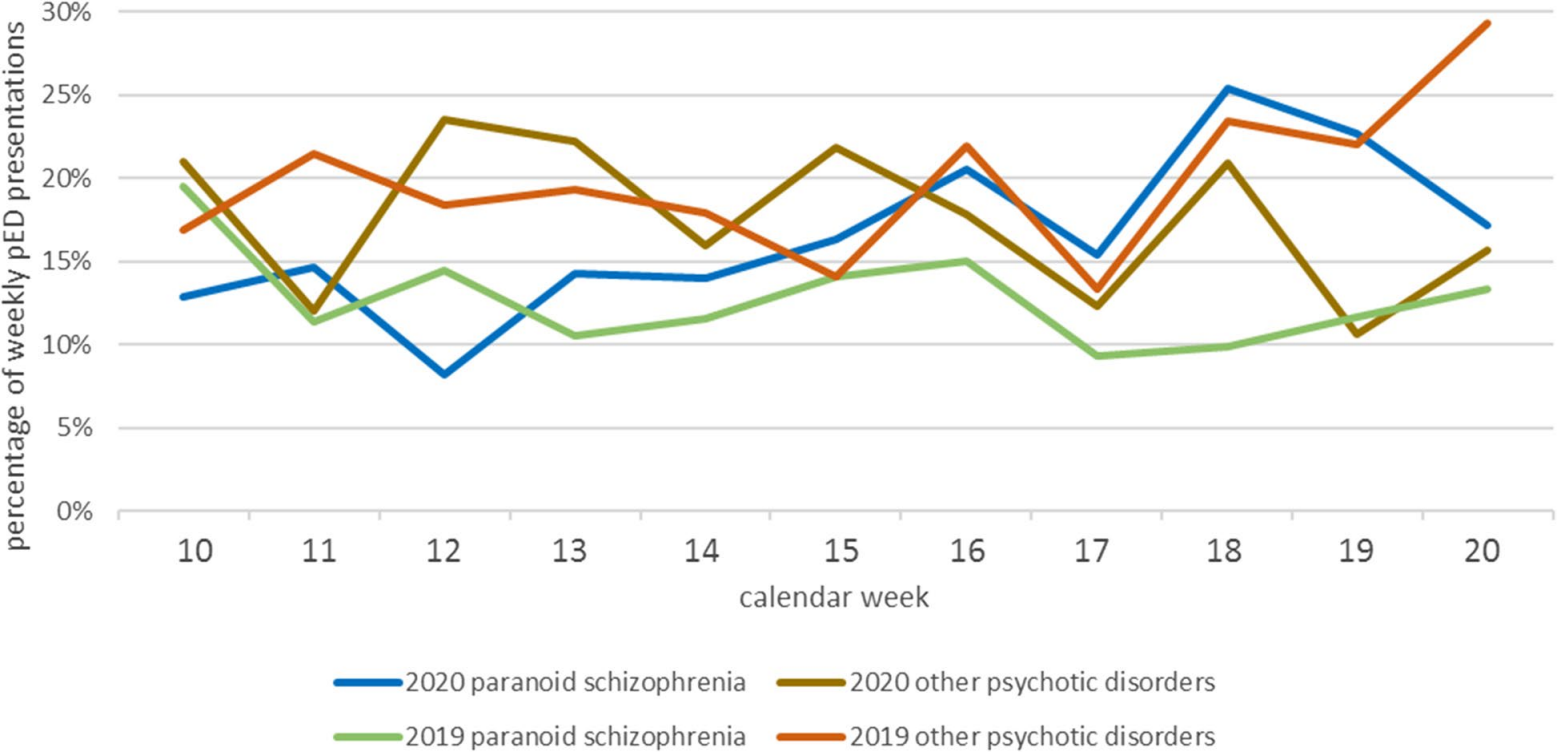
pED presentations with schizophrenia and other psychotic disorders. Displays the percentage of pED presentations with diagnoses of paranoid schizophrenia or other psychotic disorders per week per observed time period: pED presentations with paranoid schizophrenia increase over time in the Covid-19 period (blue line) but not in the pre-Covid-19 period (green line), whereas other psychotic disorders do not differ between the two observed time periods (dark brown line: Covid-19 period; light brown line: pre-Covid-19 period). Abbreviations used: *pED,* psychiatric emergency department

Presentations with schizophrenia and other psychotic disorders (SPD) did not differ during the Covid-19 period compared to the pre-Covid-19 period (Table 1). When focusing on paranoid schizophrenia alone, we could see an absolute increase of presentations during the Covid-19 period (137 vs. 115, + 19.1%, *p* = 0.020, Table 1). Paranoid schizophrenia was a predictor of Covid-19 period in the logistic regression analysis, too (Table 4). An increase in rates of presentations with SPD during the Covid-19 period was shown in various studies of similar design [12, 18, 19, 23, 49–51]. One may speculate that patients with chronic psychotic disorders and high need of psychosocial facilities have suffered more than other diagnostic groups from the lockdown restrictions which included closing or limited accessibility of many psychosocial [41] and psychotherapeutic [52] facilities. However, there are also studies, which do not show an overall increase in presentation rates in patients with SPD [15, 21, 28, 30], stressing local differences. An absolute increase in presentations with paranoid schizophrenia has only been reported in few studies [12, 51]. Interestingly, these studies have longer observation periods (6 months or longer) than most other Covid-pED studies yet. Earlier pED studies covered mostly 4 to 8 weeks. Our study has a rather long observation period with 12 weeks. In line with the finding from Jagadheesan et al. [12] the presentation rates with schizophrenia (Fig. 3) increase mainly at the end of the Covid-19 period.

It could be discussed whether this is due to weeks of strain with restrictions and Covid angst. However, a study from Ireland did not find an increase in pED presentations with psychotic disorders in their 1-year observation period [53]. Their study comprises more non-lockdown than lockdown periods, suggesting that the increase in psychotic pED presentations predominantly occurs in lockdown periods.

We observed a trend to an absolute increase in numbers of presentations with paranoid schizophrenia in police custody in the Covid-19 period compared to the pre-Covid-19 period (Table 3). This trend is in contrary to both a clinical [54] by Winkler et al. and a questionnaire-based [55] study by Kølbæk et al. that showed that patients with schizophrenia subjectively deteriorated less than other diagnostic groups during the first lockdown. One possible explanation is, that Kølbæk et al. [55] focussed on a less severely ill sample than ours as their study was based on the subjective mental state of patients who did stay in touch with the mental care system and were willing to answer several questions on the telephone, which is from clinical experience not typical for a patient presenting to the pED. As the pED presentations in the current study were mainly not self-referred (Table 1), another explanation may be a discrepancy between the subjective mental state and the evaluation of others: Winkler et al. states that although patients with schizophrenia reported less emotional strain, they formed the biggest group with crisis admissions [54]. Another explanation could be that the increase of presentations with paranoid schizophrenia was driven by newly diagnosed patients in the Covid-19 period since these were not included in the above-mentioned studies. There is some evidence from Israel [56] and Italy [57] that there was a potentially stress-induced rise in new-onset psychosis in the beginning of the pandemic. If these partially developed into schizophrenia is unknown and should be addressed in future research.

All studies, so far, found that absolute numbers of presentations with depressive disorders decreased during the first lockdown [12–15, 18–22, 26, 28, 30, 31, 48, 50, 51]. In the current study the decrease was 29.4% and hence was comparable to earlier studies (68.3% [18], 43.5% [21], 28.3% [12]). The finding from the logistic regression analysis that DD was a negative predictor for Covid-19 period fits these observations. In our sample the decrease was seen equally in severe and milder forms of depression (Table 1). These findings give reason for concern, as depressive symptoms have been described to rise in adult populations [1–3], thus revealing a discrepancy between need for treatment options and attendance of mental health services.

We found a strong decline in pED presentations by − 40.2% in the group of patients with personality disorders (PD). At the same time, PD was a negative predictor of Covid-19 period (Table 4). Many earlier studies did not mention personality disorders or reported no change [22, 30, 53]. In three south European studies a comparable important decrease has been found (− 44% [31], − 34.4% [18], − 29.6% [20]). In a study from Germany, there was an increase in pED presentations with PD during the first wave of Covid-19 [28]. The current study is the first where the number of presentations of the group of PD decreased more importantly than in all other diagnostic groups (Table 1). Interestingly, presentations with PD were the only diagnostic group in the pre-Covid-19 period who came predominantly from outside the catchment area (54.5%, Table 2s in supplementary section), suggesting a higher baseline mobility in help-seeking compared to other diagnostic groups. The restrictions in mobility during lockdown (cf introduction section) seem to have had a relevant impact on this help-seeking mobility in PD: the amount of provenance from outside our catchment area is in the Covid-19 period not higher than in other diagnostic groups (34.3%, Table 2s). In other words, the decrease in PD presentations in our sample seems to be driven by a significant drop in presentations with PD from Berlin but outside our catchment area (− 68.3%); in presentations from our catchment area the drop was only − 8.3% (Table 2s). Winkler et al. found that outpatients with PD were suffering more intensely from lockdown and social distancing measures than patients with other diagnoses [54]. This in mind, we shouldn’t simply interpret the decrease in presentations as a decrease in mental health emergencies. Table 3 indicates that albeit the important decrease in numbers of pED presentations with PD, the absolute number of presentations with PD brought by police, nevertheless, increased (+ 4.5%), suggesting a high severity. Until date, there is only very few research focusing on the fate of the vulnerable group of PD during the Covid-19 pandemic. More research is necessary.

In the logistic regression analysis, Bipolar and manic disorders (BMD) were a predictor for Covid-19 period (Table 4). If assessed, most studies report decreases in pED presentations with bipolar disorders [21, 31, 48]. Gómez-Ramiro et al. reported no statistically significant changes in pED presentation with bipolar disorder [20]. In most studies, BMD was not reported separately but as part of “mood disorders”. More research is needed to solve the question if patients with BMD were particularly at risk of deterioration during the first Covid-19 lockdown.

### Police custody

The relative and absolute increase in presentations in police custody is a main finding of this study (Table 1, Fig. 2). In our sample, this increase is based on a rise in female cases in the Covid-19 vs. the pre-Covid-19 period (67 vs. 38, + 76.3%, *p* = 0.008) with a female rate of 40.4% vs. 26.0%. In comparison, on the level of all presentations per observation period the female rate in the Covid-19 period did not differ compared to the pre-Covid-19 period (38.3% vs. 42.8%, *p* = 0.055, Table 1). There is evidence of gender-dependent lockdown effects, hinting to a more important deterioration in mental health of women than in men during the Covid-19 period [58, 59]. In our sample, self-referral significantly decreased (cf paragraph “Mode of attendance” in the Discussion), only in women (− 27.9% *p* < 0.001data in supplements, Table 1s). As women were more likely to be referred in police custody, we interpret this decrease in help-seeking in women as an expression of mental health deterioration.

Police custody may present a straight-forward characteristic suggesting acuity of cases. However, in our sample, the potential increase in pED presentations in police custody in patients with paranoid schizophrenia and with PD during the Covid-period compared to the control period does not necessarily represent an increase in disease severity. For instance, Individuals suffering from schizophrenia and from some PDs with high impulsivity traits may have had more trouble adjusting to social distancing and other restrictions, thus soliciting police interventions. The psychopathological signs of severity such as signs of delusion, aggressive behaviour towards others, suicidal thoughts, suicidal plans and suicidal attempts were, as expected, often present in the subgroup of presentations with police custody (Table 2). Differential between the Covid-19 and the pre-Covid-19 period in this subgroup were suicidal attempts prior to pED attendance, which were significantly more frequent in the Covid-19 period and reported aggressive behaviour towards others which was less often present in the Covid-19 period.

To summarize: the increase in pED presentations in police custody in the Covid-19 period compared to the pre-Covid-19 period in women, with suicidal attempts, with less aggressive behaviour and predominantly with schizophrenia or PD suggests but does not prove an increase in severity in this subgroup of psychiatric patients in the early Covid-19 era. Future studies should address the number of new-onset diseases of this subgroup. Moreover, this subgroup might deserve particular concern of mental health care services in the ongoing Covid-19 pandemic.

Hospital admission rates were, as expected, high after presentation in police custody: 62.0% during the Covid-19 period and 72.6% in the pre-Covid-19 period. I.e., the increase in presentations with police during the Covid-19 period was not and against expectation, translated into more hospital admissions, but- in the contrary- in less hospital admissions (Table 2). This raises the question: why could the need of more presumably necessary admissions not be met (cf paragraph Discharge/ hospital admission in the Discussion)? The fact that there was no increase in involuntary admissions in the Covid-19 period is reassuring in this matter (Table 2).

### Suicidality

In our sample, suicidal thought and suicide attempt prior to pED presentation are predictors of Covid-19 period (Table 4). Elsewhere, we report that overall suicidal thought, plans and suicide attempts increased during the first wave of Covid-19 and not during the second wave of Covid-19 [60]. In the subgroup of presentation in police custody, we reported an increase in presentations after suicide attempt (Table 2). Further research is necessary to further determine the reasons of this increase. As an explaining factor, an increase in disease severity may be discussed. A bias may have been introduced by a potential increase in parasuicidal behaviour in order to increase chances of inpatient treatment when bed capacities were limited [60].

### Discharge and hospital admission

We found no statistically significant change in hospital admissions (− 16.9%) in the Covid-19 period compared to the pre-Covid-19 period. Nonetheless, hospital admission was a strong negative predictor of Covid-19 period in the logistic regression analysis (Table 4). A German multi-center study found a decrease of − 30.3% [32]. As the multi-center study focuses on admissions and does not provide data on pED presentations, we cannot compare our admission rate to theirs. In our sample, total admission rate dropped from 48.9 to 44.6%. There were important differences per diagnostic group. Significantly, less presentations with substance use disorder (SUD) were eventually admitted (decrease in admission rate from 57.2 to 49.9%, Table 1). Also, a significant decrease for SPD and particularly the group of paranoid schizophrenia was observed (Table 1). In the pre-Covid-19 period 67.0% of presentations with paranoid schizophrenia led eventually to a hospital admission, in the Covid-19 period the admission rate was 49.6%.

**Fig. 4:**
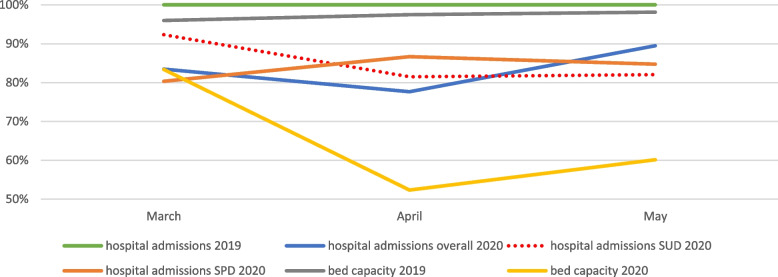
Hospital admissions (SHK) and bed capacity (SHK). Displays the psychiatric hospital admissions via the pED and bed capacity within the observed hospital in Berlin (SHK) during the Covid-19 period per month in comparison to the pre-Covid-19 period. Numbers in the pre-Covid-19 period (2019) were set to 100% for hospital admissions, i.e. for overall admissions, SUD admissions and SPD admissions (green line) . The bed capacity reached almost 100% in the pre-Covid-19 period (2019, grey line). The graph (blue line) shows that there were less overall admissions in the Covid-19 period, though the difference to the pre-Covid-19 period did not reach statistical significance. However, in the subgroup of SUD (dotted red line) and SPD (orange line) significant decreases in admissions via the pED were found. The yellow line shows a reduced bed capacity during the Covid-19 period

To understand the important differences in admission rates, it is indispensable to consider the bed capacity in these days. The bed capacity in the Covid-19 period was significantly lower (Table 1) as one of the seven wards was turned into a Covid-19 ward and other infection-avoiding guidelines were implemented (cf Study design in chapter Methods). Figure 4 shows the admissions via the pED per month in the two observation periods (the admissions in the control period are set to 100%).

Figure 4 shows that hospital admissions via the pED in our hospital did not decrease for all patients groups but for patients with SUD and SPD (with paranoid schizophrenia). The fact that overall bed capacity was clearly reduced in the Covid-19 period and overall admissions via the pED did not decrease significantly, can be explained by a significant decrease in scheduled admissions (not via the pED) during the Covid-19 period compared to the pre-Covid period (27 vs 110, data not shown). This paper is the first, taking the bed capacity into account. Its implication is that lower admission numbers during the Covid-19 period as seen in many studies and in subgroups in the current study, too, do not mandatorily mean that there was a decrease in severity of presentations, but the driving factor could be the higher admission threshold due to a lower admission capacity. The policy implemented to avoid Covid-19 infection and collaterally diminishing bed capacity was certainly well contemplated and there is by now very strong evidence that the then pandemically present Covid-19 subtypes can lead to life-threatening disease, especially in psychiatric patients [61] and infection should consequently be avoided. Additionally, we should consider that at the observation time period no vaccinations were available.

Above, we suggested with some caveats that patients presenting in police custody might represent a severely ill subgroup. However, less admissions were observed in this subgroup (admission rate decreasing from 72.6 to 62.0%, Table 2). What is more, in this subgroup (cf previous paragraph), suicide attempts prior to pED presentation were more frequent in the Covid-19 than in the pre-Covid-19 period. Together with the decrease in hospital admissions in patients with SUD and paranoid schizophrenia, this might represent rather a consequence to limited bed capacity than a reduction in required inpatient treatment.

The fact that admissions were shorter in the Covid-19 period (8 vs. 10 days, Table 1) is also most likely due to the need of creating admission capacity and not due to less treatment necessity. This shortening was driven by significantly shorter admissions for SUD, NSSD and PD (5 vs. 8, 5 vs. 12 and 3 vs. 10 days respectively, Table 3s, supplementary section).

Taken together, we conclude that a lower bed capacity (due to infection curbing measures) might has led to a higher threshold to admit pED patients for inpatient treatment. We assume that the phenomenon of limited bed capacity due to infection-avoiding policies was - at least in Germany - existent common problem during the first Covid-19 wave as there was a governmental financial support from March 16th 2020 on for leaving beds empty for prospective Covid-positive patients who were - in Germany and Nordic countries - scarce in the beginning of the pandemic (cf no Covid-positive patients in our sample).

### Strengths and limitations

We want to highlight some strengths of our study: it is based on thorough clinical documentation of which each case was reviewed individually. We covered a relatively large observation period (12 weeks) with a comparably large number of assessed pED presentations and subsequent admissions. Due to knowledge from previous studies, we were able to select the independent variables for the logistic regression model in an informed manner. This study is the first considering bed capacity.

There are also some limitations to be considered. First, it is important to point out, that this study is limited to one site, where data was collected. Furthermore, the control data is limited to the previous year only. Extrapolation of results should therefore be done cautiously. Although we implemented measures to minimize inter-rater bias during the process of data extraction from clinical records, we cannot rule out, that inter-rater bias exists to some extent. Also, there might be a bias in attention to certain details from the primary examining staff. It seems plausible, that e.g. medical conditions leading to a Covid-19 high-risk group classification were more likely to be asked for and documented during the Covid-19 pandemic. Furthermore, obesity was not always documented, only in severe cases. Also, in our sample of the beginning of the pandemic the knowledge of high-risk groups was possibly not as widely spread as later in the pandemic. Generally, the extent of documentation varied between the individual emergency ward files, possibly leading towards a more detailed description of more severe cases. Since this was rather evenly spread over the two compared time periods, we only see a minor bias risk due to this factor. Another limiting factor is the absence of a severity scale.

## Conclusion

In the Covid-19 period overall pED presentations did not change compared to the pre-Covid-19 period, whereas in diagnostic subgroups a decrease (PD and DD) or an increase (paranoid schizophrenia) in pED presentations was observed. The fact that police custody, suicidal thought and suicide attempt were predictors for Covid-19 period pED presentations suggests a higher severity in the Covid-19 cohort. However, as severity was not assessed in a standardized manner, more research is necessary on this subject. The group of patients with paranoid schizophrenia might have suffered particularly under lockdown measures as it was the only diagnostic subgroup with a rise in pED presentations in the Covid-19 period. The rise in pED presentations in police custody in the Covid-19 period was seen only in female patients and might represent gender-specific lockdown effects, which should be studied in more depth.

Hospital admission was the highest negative predictor for Covid-19 period and admission rates in SUD and SPD decreased in the Covid-19 period compared to the pre-Covid-19 period. Reduced bed capacity due to infection curbing measures is suggestive to play an important role in augmenting the threshold for hospital admissions. From infectious diseases epidemiological perspective, these measures were probably necessary in order to avoid infections, from solely psychiatric perspective the admission policy was presumably too restrictive. This caveat should be taken into account in future crises.

## Supplementary Information

**Additional file 1: S1.** Overview of excluded and merged cases. **S2.** Composition of diagnostic categories.

**Additional file 2: Table 1s.** Comparison of mode of attendance by gender presenting to the psychiatric emergency department during the first wave of Covid-19 in 2020 and the comparison period in 2019. **Table 2s.** Comparison of residency of across diagnostic categories presenting to the psychiatric emergency department during the first wave of Covid-19 in 2020 and the comparison period in 2019. **Table 3s.** Comparison of median hospital admission duration across diagnostic categories presenting to the psychiatric emergency department during the first wave of Covid-19 in 2020 and the comparison period in 2019

## Data Availability

The datasets used and analysed during the current study are available from the corresponding author on reasonable request.
