# Correction to: Psychiatric presentations and admissions during the first wave of Covid-19 compared to 2019 in a psychiatric emergency department in Berlin, Germany: a retrospective chart review

**DOI:** 10.1186/s12888-023-04629-8

**Published:** 2023-03-14

**Authors:** T. Goldschmidt, Y. Kippe, A. Finck, M. Adam, H. Hamadoun, J. G. Winkler, F. Bermpohl, M. Schouler-Ocak, S. Gutwinski

**Affiliations:** Psychiatrische Universitätsklinik der Charité im St. Hedwig Krankenhaus, Große Hamburger Str. 5-11, 10115 Berlin, Germany

Following publication of the original article [1], the authors identified an error in the author name of S. Gutwinski.

The incorrect author name is: S. Gutwinksi

The correct author name is: S. Gutwinski

The author group has been updated above and the original article [1] has been corrected.
