# Supplementary material for: Psychiatric presentations and admissions during the first wave of Covid-19 compared to 2019 in a psychiatric emergency department in Berlin, Germany: a retrospective chart review
Source: BMC Psychiatry. 2023 Jan 14;23:38. doi: 10.1186/s12888-023-04537-x (PMC9839445; doi:10.1186/s12888-023-04537-x)
Supplement: Supplementary file 1 — Additional file 1: S1. Overview of excluded and merged cases. S2. Composition of diagnostic categories. [file 12888_2023_4537_MOESM1_ESM.docx]

**Supplementary Material**

**S1 Overview of excluded and merged cases**

**N = 2314** cases during the 2 observation periods

**N = 2304** cases after excluding duplicate clinical records

**N = 1936** cases after exclusion criteria

**N = 1840** cases included after merging cases

**n = 10** cases with duplicate clinical records

**n = 17** somatic cases

**n = 28** consultations in preparation of a planned hospital admission

**n = 137** scheduled admissions (waiting list procedure)

**n = 174** day therapy cases (patients receive treatment in hospital but sleep in their own home)

**n = 3** consultations as follow-up after hospital discharge

**n = 8** cases without clinical documentation

**n = 1** cases with non-medical issues (e.g.: patients in need of shelter)

**n = 96** cases merged:

**n = 71** cases with *0-3 days* interruption of hospital admission

**n = 13** cases with *4-7 days* interruption of hospital admission

**n = 12** visits of emergency department, in between two merged hospital admissions

**N = 1707 cases included in analysis**

**n = 133** cases left without being seen by medical/psychiatric staff

**S2 Composition of diagnostic categories**

Organic mental disorders (OMD): F00 – F09

Substance use disorders (SUD): F10 – F19

Not included: F17 nicotine/tobacco related substance use disorders

F1x.5 substance induced psychotic disorder

F1x.7 late-onset substance induced psychotic disorder

Schizophrenia and psychotic disorders (SPD): F20 – F29

F1x.5 substance induced psychotic disorder

F1x.7 late-onset substance induced psychotic disorder

Bipolar and manic disorders (BMD): F30 – F31

Depressive disorders (DD): F32 – F33

Neurotic-, somatoform and

stress-related disorders (NSD): F40 – F48

Personality disorders (PD): F60 – F62
